# Assessment of acute toxicity and developmental transformation impacts of polyethylene microbead exposure on larval daggerblade grass shrimp (*Palaemon pugio*)

**DOI:** 10.1038/s41598-022-10999-y

**Published:** 2022-04-28

**Authors:** Austin D. Gray, John E. Weinstein, Rachelle C. Riegerix

**Affiliations:** 1 Department of Biological Sciences, Virgina Polytechnical Institute and State University, 926 W Campus Dr, Blacksburg, VA 24060 USA; 2 Department of Biology, The Citadel, Military College of South Carolina, 171 Moultrie Street, Charleston, SC 29402 USA; 3 U.S. Environmental Protection Agency, Office of Land and Emergency Management, Office of Resource Conservation and Recovery, William Jefferson Clinton West Building (WJC West), 1301 Constitution Avenue N.W., Washington, DC 20004 USA

**Keywords:** Ecology, Ecology, Environmental sciences

## Abstract

Due to the ubiquity of microplastic contamination in coastal waters, there is potential for adverse impacts to organism development. One organism of interest is the daggerblade grass shrimp, *Palaemon pugio*, an ecologically important species in estuaries along the east coast of North America. We exposed larval grass shrimp to virgin polyethylene microbeads (35 and 58 µm) at a high (0.375 and 1.95 mg/L), medium (0.0375 and 0.195 mg/L), and a low concentration (0.00375 and 0.0195 mg/L), respectively for 23 days to assess mortality, transformation time from larval to juvenile stage, and weight. Average percent mortality was 3.7 to 4.8 times higher in the experimental treatments compared to controls. The greatest proportion of mortality was observed in the first 11 days. Median time for transformation ranged from 20.2 to 20.8 days. Shrimp exposed to the 35 µm beads in the high treatment (20.2 days) transformed significantly faster than the control shrimp (20.8 days). Although development was not delayed and size of the shrimp did not differ, the acute toxicity of microplastics on grass shrimp is a concern due to their role in energy cycling within tidal-creeks. These findings suggest potential population and community level effects following microplastic exposure.

## Introduction

The presence of microplastic particles in coastal waters has been a growing concern over the past decade due to their potential adverse impacts on the health of wildlife and humans alike, review by Ref.^1^. Although sources of these particles vary and may be site specific, several studies have suggested that the degradation of macroplastic litter, producing secondary microplastics, is an important contributor^2–5^. Microplastic particles in coastal waters can also originate from commercial sources where the plastic particle is intentionally manufactured to be microscopic (primary microplastic)^6^. Regardless of source, the overall abundance of microplastics in coastal areas can vary depending on whether researchers sample sediments or surface water. Microplastic concentrations reported in coastal sediments range between 0 to 8720 particle/kg^7,8^, and in coastal waters range between 0.002 to 468,00 particles/m^3^ (0 to 468 particles/L)^7,9,10^. Sizes of microplastics detected in coastal waters range from the nano to micrometer scale^1,11^, with varying classifications (fragment, fiber, bead, and tire wear particle)^12,13^. The presence of microplastics in the environment is likely to increase due to the ongoing increases in plastic production, with a total global production of 368 M tonnes in 2019^14^.

Due to their small size, microplastics can be ingested by a wide range of organisms including fish, crustaceans, and zooplanktonic organisms^15–19^, filter-feeding and suspension-feeding organisms, such as mussels (*Mytilus edulis*)^20–22^, oysters (*Crassostrea virginica*)^23^, and copepods (*Tigriopus japonicus*)^24,25^ because their size range overlaps with that of common prey items^26^. While the previously mentioned studies reported microplastics in biota, laboratory studies have aimed to understand the toxicological impacts of microplastics to assess endpoints such as growth, reproduction, and mortality^17,27^. Gray and Weinstein^17^ attributed toxicity to the size and shape of the particle, suggesting that the physical attributes of the particle are an important determinant in the toxicological response. Because microplastic particles exhibit a large surface-area-to-volume ratio, it is also possible that the toxicological responses observed in the lab may differ with the use of weathered or secondary microplastics due to the leaching of additives and sorbed environmental contaminants from the particles^28^. In any case, it is important to consider the toxicological responses to both virgin (commercially produced and not previously exposed to environmental conditions) and weathered (environmentally-derived) microplastic particles to discern toxicity due to physical attributes of the particles from those that may be due to the leaching of additives and sorbed contaminants.

Aquatic invertebrates, specifically crustaceans, play a key role in maintaining the health of their respective habitats. Any adverse effect of microplastics on these organisms may have impacts at the ecosystem level. Along the east coast of North America, grass shrimp (*Palaemon *spp.) are an ecologically important estuarine species, playing a vital role in energy cycling within tidal creek-salt marsh habitats^29^. Grass shrimp account for ~ 83% of the total nekton biomass^30^. Grass shrimp are consumed by various predators^31^, including commercially and recreationally important species of seafood. Given their epibenthic lifestyle within these tidal creek-salt marsh systems, grass shrimp are likely to encounter relatively high levels of microplastic particles. And, because of their key role in salt marsh habitats, negative implications of the microplastic exposure to this species could include decreased biodiversity and impacts on commercially and recreationally important fisheries^32^.

An abundance of microplastics present in the environment are in surface waters, where zooplankton such as larval grass shrimp feed, increasing the chances of exposure and ingestion^33^. Gray and Weinstein^17^ demonstrated that microplastic fragments, fibers, and beads can influence adult grass shrimp mortality via ingestion following an acute or short-term exposure (3 h). Leads et al.^34^ found that microplastic ingestion did not alter grass shrimp susceptibility to bacterial infection (*Vibrio campbellii*). These findings demonstrate that microplastic exposure can have varying effects depending on the end-point measured (mortality vs immune response). However, there is no information about the impact microplastics may have on the transformation of grass shrimp from the larval stage to the juvenile stage. Early developmental stages of organisms exhibit a higher sensitivity to toxicants than other life stages, which may consequently adversely affect population levels^35^. The impact of microplastics on the development of aquatic organisms is not as well-understood as other toxicity endpoints, such a mortality, immune response, and gene expression^36^. Particles less than 100 µm are of interest since in the natural environment, microplastic particles less than 100 µm is more abundant than those larger than 100 µm ^37^. Gray and Weinstein^17^ found that there was low mortality (0 to 10%) when adult grass shrimp were exposed to polyethylene (PE) microbeads less than 100 µm (35 and 58 µm). Due to their low mortality to adults, those particle sizes were used to investigate whether PE microbeads impacted larval grass shrimp development. Given the ecological importance of the *Palaemon* spp. for salt marsh ecosystems and the vulnerability of coastal habitats to anthropogenic disturbance, it is important to understand how microplastics may impact their development*.*

In the present study, we exposed larval grass shrimp (2 days post hatch) to virgin PE microbeads. PE is one of the most commonly used plastics globally, and consequently the most encountered polymer type in marine environments^38^. Size fractions used were the least toxic to adult grass shrimp based on previous work conducted by Gray and Weinstein^17^. We aimed to assess if exposure to virgin PE microbeads at a low, medium, and high concentration with two different particle sizes would result in acute toxicity or have any effect on the transformation of grass shrimp from larval to juvenile stage.

## Materials and methods

### Grass shrimp collection

Gravid adult grass shrimp were collected from Leadenwah Creek, Wadmalaw Island, South Carolina (USA; 32° 38′, 50.6″ N, 80° 13′,14.0″ W) with a dip net. Leadenwah Creek is a tidal tributary not impacted by urban disturbance. This site also serves as a long‐term ecological monitoring reference site^39^, and organisms collected from this site have been used in previous laboratory assays^17,34,40^. Gravid shrimp with embryos in stage VI (oval eyespots, rapid heartbeat)^41^ were transported to the Aquatic Toxicology Laboratory at The Citadel, Military College of South Carolina (Charleston, SC, USA). Shrimp were held at 24 °C in aquatic tanks (~ 19 L) containing filtered seawater from Charleston Harbor with an 8 h light:16 h dark photoperiod. Gravid shrimp that were expected to hatch within 2 days post collection were placed into an enclosed mesh cage within a separate tank (~ 19 L). Once hatched, the larval shrimp were collected using a dip net and transferred to a separate holding tank (~ 19 L). Larval shrimp were allowed to acclimate for 48 h prior to microbead exposure. Laval grass shrimp can survive for ~ 10 days without food but can only transition to the second larval instar^42,43^. As a result, shrimp larvae were fed brine shrimp nauplii twice daily during their acclimation period and throughout the developmental assays^44^. Foundational work published by Ref.^43^ demonstrated the ability of larval grass shrimp to consume nauplii two days post hatch. Larval shrimp are typically 2.6 mm in length after hatching but can range between 6 to 8 mm in length at metamorphosis^45,46^. Mean brine shrimp nauplii length and width ranges between (517 to 618 µm) and (144 to 177 µm), respectively^48^.

### Developmental assays

To assess the effect of PE microbead exposure on larval grass shrimp development, two assays using different particle sizes (sizes 32–38 and 53–63 µm; 1.02 to 1.03 g/cc) were conducted with fluorescent green PE microbeads purchased from Cospheric (Santa Barbara, CA). Microbead sizes will be referred to by the median size fraction (35 and 58 µm), hereafter. Microbeads were purchased in dry form and did not come in any detergent or antimicrobial solutions. Each assay consisted of a low (62.5 particles/L), medium (625 particles/L), and high (6250 particles/L) treatments. Control (no microbead) solutions were also concurrently tested. Treatment concentrations reflected both relevant and above relevant microplastic concentrations in nature. Particle concentrations were estimated based on weight. For the 35 µm treatment the low, medium, and high treatments were 0.00375, 0.0375, and 0.375 mg/L, respectively. For the 58 µm treatment the low, medium, and high treatments were 0.0195, 0.195, and 1.95 mg/L, respectively. Since we used three concentrations that increased on a log scale we classified them as low, medium, and high treatments. Hereafter, concentrations will be reported by designated classification (low, medium, and high.

Test chambers (glass beakers; 250 mL) contained 15 larval shrimp and 200 mL of solution. There were three replicate chambers per treatment and two replicate chambers for the control treatments. Since each replicate had the same number of grass shrimp, this allows for comparisons to be made between control and experimental treatments. Slight aeration (~ 1 bubble per second) was used to ensure microbeads remained suspended throughout the solution. Water changes (100%) occurred every 48 h with new microbeads added to each respective treatment. Water quality was monitored and recorded every 2 days prior to water changes. Dissolved oxygen, pH, and salinity were 7.76 ± 0.42 mg/L, 7.8 ± 0.2, and 28.7 ± 1.6 ‰, respectively (mean ± SD). Temperature was held at a constant 24 °C. Shrimp were fed a diet of newly hatched Artemia nauplii (1 drop into each chamber) once daily^29^.

Assays began when larvae were 2 days post-hatch, and the exposure continued until 90% of the shrimp larvae successfully transformed to the juvenile stage, which typically occurs between 15 and 22 days in the laboratory^48^. Assays were terminated when > 90% of the shrimp larvae successfully transformed to the juvenile stage. Determination that shrimp had transformed was based phenotypic observations designated by the transition from upside down and backwards swimming during the larval stage to dorsal side up and forward swimming in the juvenile stage, we did not assess molting stages and number of molts to reach the juvenile state^49,50^. The number of larvae successfully transforming were monitored daily. Mortality was monitored at each water change. At the conclusion of the study, juvenile shrimp were air dried overnight and weighed individually on a microbalance.

### Statistical analysis

The median time to transformation was determined using probit analysis followed by the LC50 ratio test to test for differences among treatments^51^. Differences in shrimp weight at the end of the experiment and percent mortality were assessed using a Kruskal–Wallis Test with a Pairwise Wilcoxon post-hoc analysis. All analyses were conducted using SAS Enterprise 4.3 and R Studio v3.5. All figures were made in GraphPad Prism v9.2.0.

## Results

**Figure 1:**
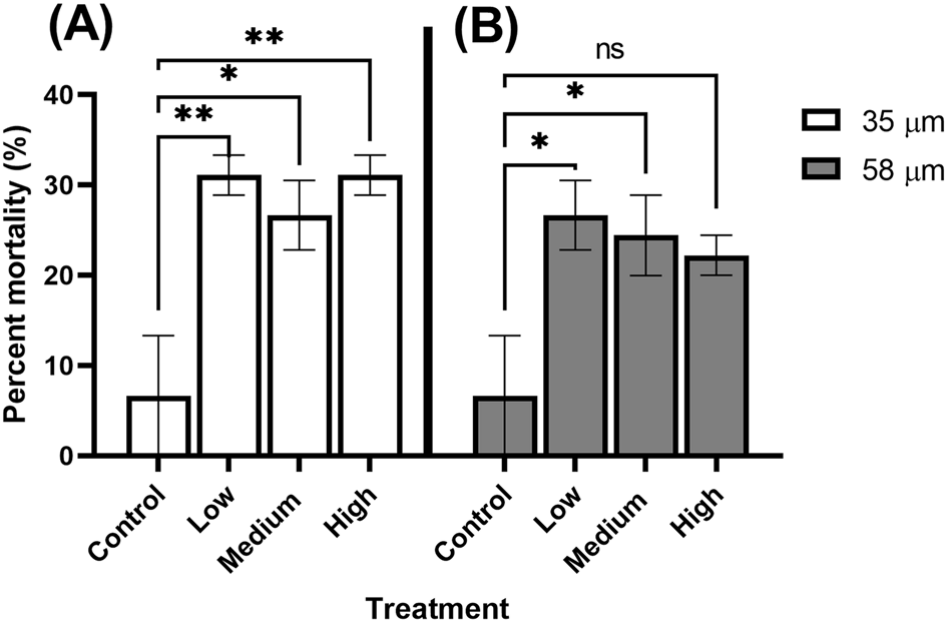
Percent mortality of shrimp from size treatments. (**A**) 35 µm treatment and (**B**) 58 µm treatment. Experimental treatments were statistically compared to control treatments. *Denoted p-value that is < 0.05. **denotes p-values that is < 0.01. ns = not significant.

**Figure 2:**
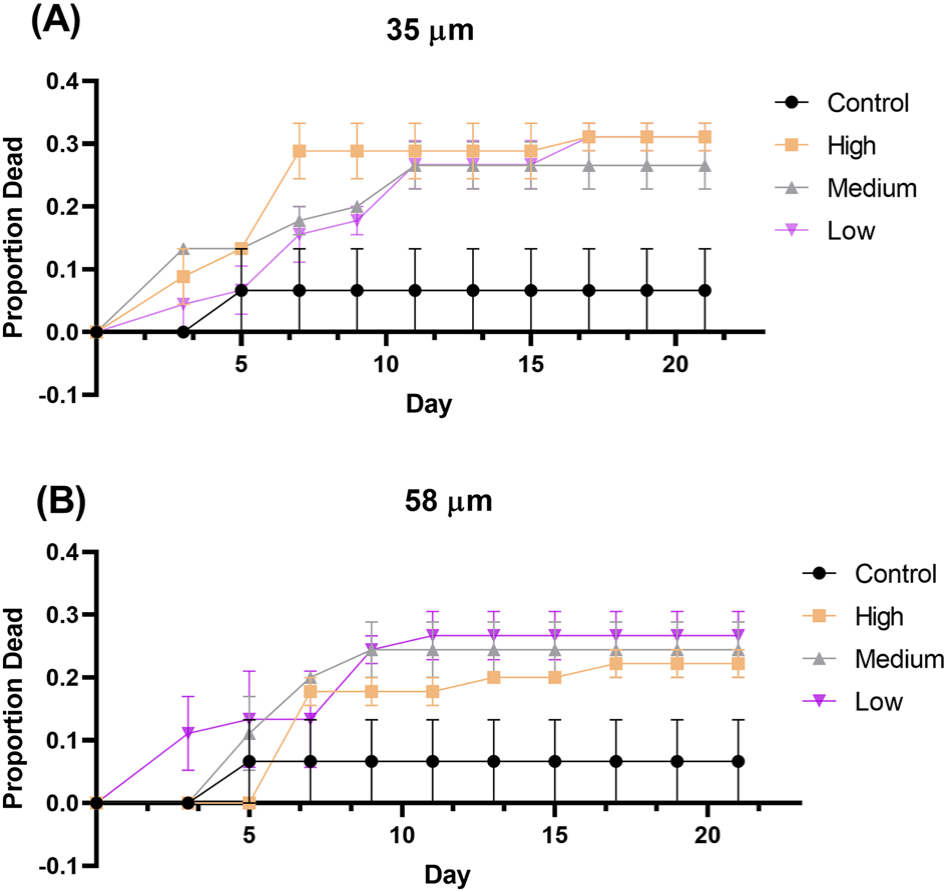
Toxicity curve graph showing the proportion of shrimp that died over the 23-day exposure relative to the initial concentration. (**A**) Depicts the proportion from the 35 µm exposure and (**B**) depicts the proportion from the 58 µm exposure.

**Figure 3:**
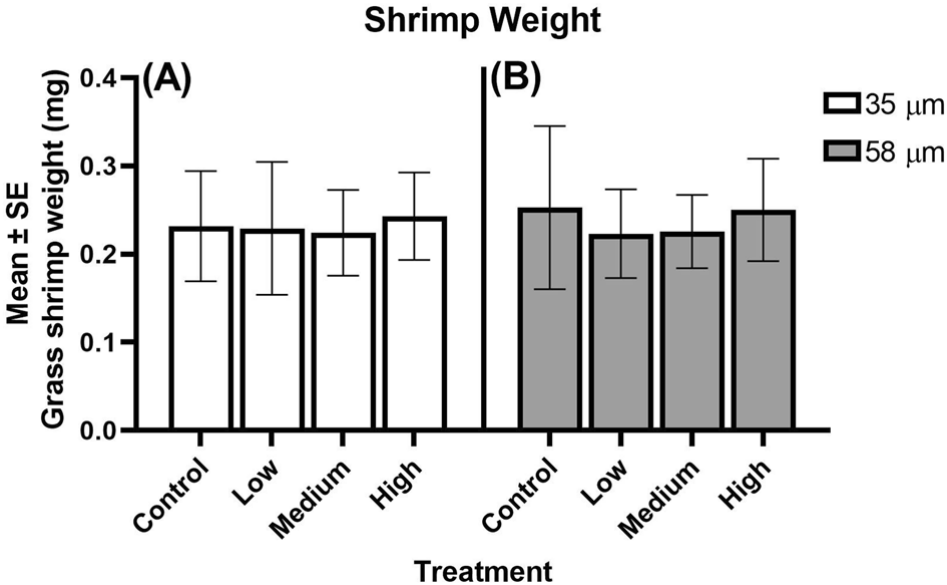
Mean weights of shrimp from size treatments. (**A**) the 35 µm exposure and (**B**) the 58 µm exposure along with control weights. We observed no difference in shrimp weights among the three treatments and the control for both particle sizes.

For the 35 µm microbead experiment, average mortality from the experimental treatments was 4 to 4.5 times higher than the control treatment (6.7%) (p = 0.01). Average percent mortality from the low, medium, and high treatments was 31.1%, 26.7%, and 31.1%, respectively (Fig. 1A). Percent mortality in each treatment was significantly higher than the control (p < 0.01 in each treatment; Fig. 1A). Mortality was highest during the first half of transformation time (0–11 days) (Fig. 2A). Larval transformation to the juvenile stage was first evident on Day 19 in the control and all experimental treatments. Median transformation time was significantly faster in the high treatment (20.2 days [20.0–20.5 days]) (medial transformation time [95% Confidence Interval]) relative to the control (20.8 days [20.5–21.0 days]) (Table 1). Median transformation times in the low and medium treatments ranged from 20.6 to 20.9 days but did not significantly differ from that of the control (Table 1). Mean juvenile shrimp weights ranged from 0.219 ± 0.041 mg in the controls to 0.231 ± 0.041 mg in high treatment (Fig. 3A). Despite there being larger shrimp in the high treatment, there was no significant difference in weights among the various treatments at the conclusion of the experiment on Day 23 (Fig. 3A, χ^2^ = 3.43, DF = 3, p = 0.33).

**Table 1:** Median transformation time (MTT) and 95% confidence intervals for the 35 and 58 µm polyethylene microspheres treatments.

Treatment	MTT (days)—35 µm	MTT (days)—58 µm
Control	20.8 days (20.5–21.0 days)	20.8 days (20.5–21.0 days)
Low	20.6 days (20.3–20.9 days)	20.5 days (20.3–20.7 days)
Medium	20.9 days (20.6–21.2)	20.5 days (20.3–20.7)
High	20.2 days (20.0–20.5 days)*	20.6 days (20.4–20.8 days)

*Denotes transformation treatment that was significantly faster than the control.

For the 58 µm microbead experiment, experimental treatment exhibited percent mortality that was 3.3 to 3.9 times higher than the controls. The control mortality was the same as what was observed in the 35 µm study (Fig. 1B). Percent mortality in the experimental treatments was generally lower than that observed in the 35 µm size fractions with the low, medium, and high average mortality being 26.7%, 24.4%, and 22.2%, respectively (Fig. 1B). However, the percent mortality from the low and medium treatments was significantly higher than the control (p < 0.01, p < 0.01; Fig. 1B). As observed in the 35 µm treatments, mortality was highest in the first 11 days of the exposure (Fig. 2B). Similar to the larval shrimp in the 35 µm treatment, larval transformation to the juvenile stage was first evident on Day 19 across all treatments. There was no significant difference in median transformation time among the three treatments relative to the controls (p > 0.05; Table 1). The median time to transformation ranged from 20.5 days [20.3–20.7 days] in the low and medium treatments to 20.8 days [20.5–21.0 days] in the control (Table 1). Mean shrimp weights ranged from 0.221 ± 0.034 mg in the medium treatment to 0.244 ± 0.054 mg in the high treatment (Fig. 3B). Similar to the 35 µm study, we observed no significant difference in shrimp weight at the conclusion of the experiment on day 23 (Fig. 3B; χ^2^ = 4.23, DF = 3, p = 0.24). When comparing mortality between the 35 and 58 µm size fractions, we observed no difference among treatment groups (p > 0.05).

## Discussion

We did not find any evidence that the presence of virgin PE microbeads in either the 35 or 58 µm size ranges experimental treatments delayed the developmental transition of the larval grass shrimp to the juvenile state. It is important to note that medium and high concentrations used in the study were higher than those reported in coastal surface waters of South Carolina (7 to 31 microplastic particles/L)^2^, with the low concentration being more environmentally relevant and comparable with levels of microplastics reported in coastal waters of Portugal, Australia, Japan, and Indonesia^9,52–54^. However, the concentration used in the medium treatment (625 particles/L) was comparable to levels reported by Hosseini et al.^10^ where average surface water concentrations ranged between 86 to 362 particles/L, and the dominant particle type was PE. The high treatment (6250 particles/L) concentration of particles are not representative of environmental levels reported in coastal waters but is comparable to levels reported in stormwater, sweepsand, and washwater (~ 4500 particles/L)^55^, indicating levels used in the present study can be found in the environment.

Exposure to both size fractions of microbeads were acutely toxicity to larval grass shrimp, with mortality ranging from 22 to 31% (Fig. 1). Although percent mortality did not differ between the two size fractions, there were higher mortality reported for the 35 µm size fraction exposures. By contrast, Gray and Weinstein^17^ reported < 10% mortality in adult grass shrimp exposed to PE microbeads of the same size fraction used in the present study, with 35 µm particles exhibiting no mortality^17^. These results suggest that PE microbeads are more toxic to larval grass shrimp than adults. Relatively higher sensitivity to toxicant exposure for larval grass shrimp compared to adults has also been reported for malathion^56^, permethrin^57^, chlorothalonil^58^, and PBDE^59^. Our results demonstrate that sensitivity to toxicants like microplastics can depend on the age of the organisms. However, this does not necessarily mean that adverse effects such as delayed development, reduced growth and emergence as observed by Ziajahormi et al.^60^ and Redondo-Hasselerharm et al.^61^ cannot be seen with other sediment dwelling invertebrates. The greatest mortality was observed over the first half of exposure (day 0 to day 11) (Fig. 2). There may be a specific time range in development where sensitivity to contaminants like microplastics is greater based on our results. This warrants further investigation. We did not observe any microbeads being ingested by the shrimp that may have influenced mortality as seen in Gray and Weinstein^17^. It is possible these microplastics caused a stress response that caused irreversible cellular damage resultng in mortality, as observed by Lehtiniemi et al.^62^.

Findings from the present study highlight that microbead exposure in aquatic habitats does not necessarily mean that organism development is delayed. Studies having reported adverse effects on development in other organisms utilized concentrations higher than those used in the present study. For example, C. Martínez-Gomez et al.^63^ found that for sea urchin (*Paracentrotus lividus*) development, the highest percentages of larval development abnormalities were at the highest concentration (5 g of high-density PE particles/L). By contrast, the highest concentrations used in the current study were 0.375 mg/L and 1.95 mg/L for the 35 µm and 58 µm microbead assays, respectively. Our findings are consistent with previous studies reported in the literature that used virgin microbeads that resulted in no adverse impact on organism development. For example, Khosrovyan and Kahru^64^ found that virgin polyamide microplastics at environmentally relevant concentrations had no adverse effect on development and growth of the harlequin fly, *Chironomus riparius*. Similarly, Le Bihanic^65^ found that embryos and larvae of the ricefish, *Oryzias melastigma* demonstrated no adverse effects on survival, hatching, development, and behavior when exposed to virgin microplastics. Lastly, LeMoine et al.^66^ found that exposure of the early life stage zebrafish (*Danio rerio*) to PE microplastics resulted in no detrimental effects of these particles on larval development, growth, or metabolism.

In comparison to those studies using virgin microplastics, Le Bihanic^65^ found that *Oryzias melastigma* embryos and larvae exposed to perfluorooctanesulfonic acid (PFOS), benzo[a]pyrene (BaP), or benzophenone-3 (BP3) sorbed to microplastics resulted in decreased embryonic survival, prevented hatching, reduced growth, increased developmental anomalies and abnormal behavior. Collectively, these results suggest that microplastics by themselves may not be solely responsible for developmental effects. Instead, contaminants sorbed to the surface of microplastics may be more influential. A similar claim was made by Leads et al.^34^ regarding the acute toxicity of virgin microplastics on adult grass shrimp. This suggests that the hazards posed by virgin microplastics may be different compared to those microplastics present in the environment. From an experimental perspective, virgin microplastics are ideal to use because they are free of sorbed environmental contaminants, and any chemical additives (e.g. plasticizers) that may leach may be identified from technical specifications provided by the manufacturer. Also, important to note, is that 65% of studies that have assessed microplastic effects on organisms have utilized PE microbeads ≥ 10 µm^36^. Utilizing virgin microplastics allows researchers to determine the effect of the microplastic particle shape, size, and polymer composition alone without confounding factors of sorbed contaminants and/or leaching chemical additives.

Of note was the significantly faster median transformation time in larval grass shrimp exposed to the high treatment of 35 µm microbeads. A similar trend was observed, albeit not significant, for the 58 µm microbeads, as well as during preliminary microbead exposure trials (unpublished data). Freeman^67^ found that the nutritional state (larvae continually fed or fed on the first two days of the molt cycle) resulted in larger growth, indicating that nutritional state is a strong regulator of tissue growth. Although there was no significant difference in weight among shrimp, our results regarding faster transformation time observed in the high 35 µm treatment may be explained by the higher mortality seen in that treatment that possibly afforded greater food availability to shrimp that survived as they did not have to compete with more individuals for resources. A follow up to this work would be the inclusion of controlled feeding rates in to the design to determine if transformation is a result of feeding or plastic exposure. Future work investigating this phenomenon is necessary moving forward as individuals may not grow larger due to microplastic exposure but will develop faster as a result of microplastics, potentially causing community level effects (altered ecosystem function and organic matter cycling) when considering the role grass shrimp play as detritivores in estuarine habitats. Microplastics affect individuals, then the population, before finally disrupting community structure and function^68^.

## Conclusion

In the present study, chronic exposure to virgin PE microbeads did not delay development or result in juvenile weight differences for grass shrimp. Microplastics were acutely toxic to larval shrimp with up to 31% mortality observed. Due to the ubiquitous presence and continuous release of microplastics into coastal waters, populations of grass shrimp that decline due to exposure can disrupt community structure and ecosystem functions of key ecological processes (energy and organic matter cycling) in salt-marsh habitats. Although our assessment focused on virgin microplastics, environmental plastics that are weathered contain various contaminants sorbed to their surface. Consequently, future research should be cautious of how they interpret their findings when using weathered plastics instead of virgin, as effects from weathered plastics may be largely influenced by co-contaminants sorbed to the plastics. Assessments that utilize virgin plastics are useful for understanding the ecological implication of microplastic pollution as a single entity without having to consider co-contaminant exposures.

## Data Availability

Basicdata are publicly available on FigShare at Development_Study_Raw_Data: https://figshare.com/articles/dataset/Developmental_Study_raw_data/17041550, https://figshare.com/articles/dataset/Developmental_Study_raw_data_2_xlsx/18516188/1.
